# Supplementary material for: Altered Expression of the MEG3, FTO, ATF4, and Lipogenic Genes in PBMCs from Children with Obesity and Its Associations with Added Sugar Intake
Source: Nutrients. 2025 Aug 2;17(15):2546. doi: 10.3390/nu17152546 (PMC12348735; doi:10.3390/nu17152546)
Supplement: Supplementary file 1 [file nutrients-17-02546-s001.zip › Supplementary Figure 1.pdf]

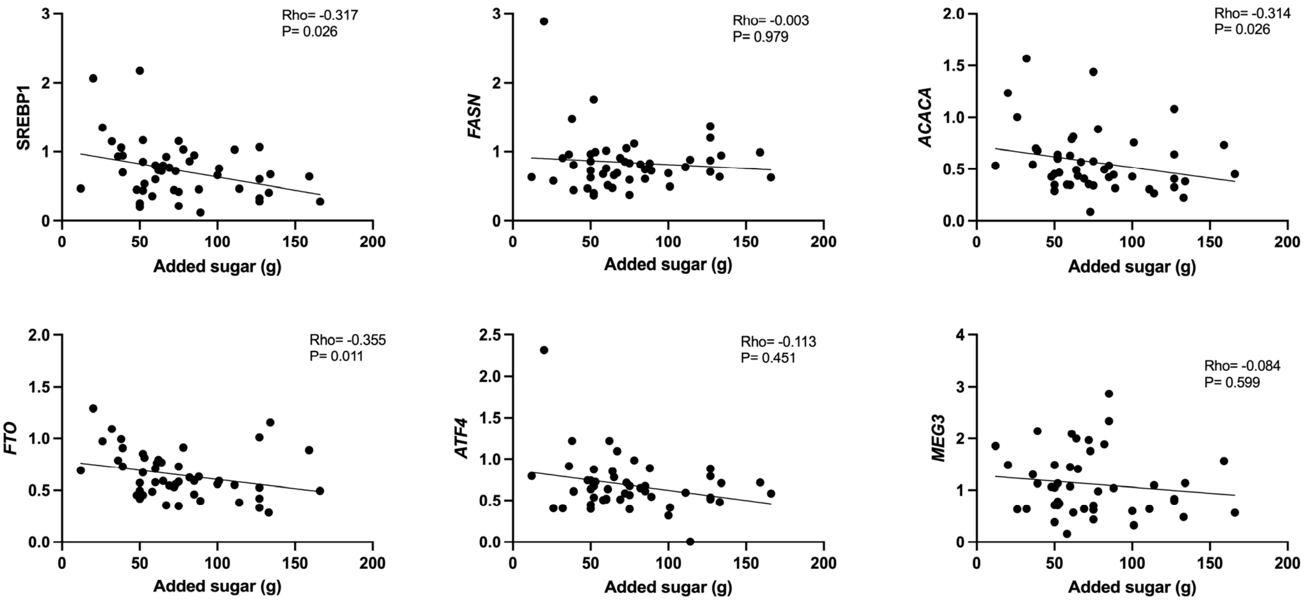

Supplementary Figure S1. Spearman's correlation coefficients between added sugar intake and lipogenic genes. Rho values correspond to the Spearman correlation coefficients. P-values <0.05 were regarded as statistically significant. *SREBP1*: Sterol Regulatory Element-Binding Protein 1; *FASN*: Fatty Acid Synthase; *ACACA*: Acetyl-CoA Carboxylase Alpha; *FTO*: Fat Mass and Obesity-Associated Gene; *ATF4*: Activating Transcription Factor 4; *MEG3*: Maternally Expressed Gene 3.
