# Supplementary material for: Altered Expression of the MEG3, FTO, ATF4, and Lipogenic Genes in PBMCs from Children with Obesity and Its Associations with Added Sugar Intake
Source: Nutrients. 2025 Aug 2;17(15):2546. doi: 10.3390/nu17152546 (PMC12348735; doi:10.3390/nu17152546)
Supplement: Supplementary file 1 [file nutrients-17-02546-s001.zip › Supplementary Table 1.pdf]

Supplementary Table S1. Spearman's correlation coefficients between *MEG3* and lipogenic genes

**Gene**	**Rho**	**P-value**
SREBP1	**0.408**	**0.001**
FASN	**0.238**	**0.025**
ACACA	0.130	0.307
FTO	**0.389**	**0.001**
ATF4	**0.422**	**< 0.001**

SREBP1: Sterol Regulatory Element-Binding Protein 1; *FASN*: Fatty Acid Synthase; *ACACA*: Acetyl-CoA Carboxylase Alpha; *FTO*: Fat Mass and Obesity-Associated Gene; *ATF4*: Activating Transcription Factor 4. Rho values correspond to the Spearman correlation coefficients. P values < 0.05 were considered statistically significant.
