# Supplementary material for: Altered Expression of the MEG3, FTO, ATF4, and Lipogenic Genes in PBMCs from Children with Obesity and Its Associations with Added Sugar Intake
Source: Nutrients. 2025 Aug 2;17(15):2546. doi: 10.3390/nu17152546 (PMC12348735; doi:10.3390/nu17152546)
Supplement: Supplementary file 1 [file nutrients-17-02546-s001.zip › Supplementary Table 2.pdf]

Supplementary Table S2. Spearman's correlation coefficients between lncRNA *MEG3*, lipogenic genes and clinical and biochemical parameters

Variable	*SREBP1c*		*FASN*		*ACACA*		*FTO*		*ATF4*		*MEG3*	
	**Rho**	**P-value**	**Rho**	**P-value**	**Rho**	**P-value**	**Rho**	**P-value**	**Rho**	**P-value**	**Rho**	**P-value**
BMI percentile	-0.194	0.107	-0.084	0.469	-0.347	0.003	-0.452	< 0.001	-0.383	< 0.001	0.069	0.585
Waist circumference (cm)	-0.223	0.062	-0.023	0.852	-0.408	< 0.001	-0.497	< 0.001	-0.340	0.003	0.029	0.816
Hip circumference (cm)	-0.185	0.124	-0.002	0.981	-0.307	0.009	-0.466	< 0.001	-0.293	0.012	0.020	0.874
Glucose (mg/dL)	-0.260	0.029	0.304	0.012	-0.187	0.117	-0.074	0.536	0.137	0.253	-0.006	0.956
Insulin **(**µIU/ml**)**	-0.354	0.003	0.223	0.073	-0.425	< 0.001	-0.426	< 0.001	-0.267	0.026	-0.168	0.193
HOMA-IR	-0.373	0.001	0.254	0.040	-0.438	< 0.001	-0.419	< 0.001	-0.241	0.046	-0.193	0.135
Total CHO (mg/dL)	-0.071	0.558	-0.082	0.507	0.105	0.381	-0.064	0.592	-0.078	0.517	0.110	0.289
HDL-C (mg/dL)	0.046	0.701	-0.168	0.172	0.302	0.010	0.202	0.090	0.127	0.288	-0.066	0.606
LDL-C (mg/dL)	-0.042	0.725	-0.024	0.843	0.163	0.174	-0.010	0.929	-0.119	0.320	0.090	0.479
TG (mg/dL)	-0.039	0.743	0.090	0.465	-0.276	0.019	-0.209	0.079	-0.077	0.518	0.036	0.774

BMI: Body Mass Index; HOMA-IR: Homeostatic Model Assessment of Insulin Resistance; CHO: Cholesterol; HDL-C: High-Density Lipoprotein Cholesterol; LDL: Low-Density Lipoprotein Cholesterol; TG: Triglycerides; *SREBP1*: Sterol Regulatory Element-Binding Protein 1; *FASN*: Fatty Acid Synthase; *ACACA*: Acetyl-CoA Carboxylase Alpha; *FTO*: Fat Mass and Obesity-Associated Gene; *ATF4*: Activating Transcription Factor 4; *MEG3*: Maternally Expressed Gene 3. Rho values correspond to the Spearman correlation coefficients. P values < 0.05 were considered statistically significant.
