# Supplementary material for: Altered Expression of the MEG3, FTO, ATF4, and Lipogenic Genes in PBMCs from Children with Obesity and Its Associations with Added Sugar Intake
Source: Nutrients. 2025 Aug 2;17(15):2546. doi: 10.3390/nu17152546 (PMC12348735; doi:10.3390/nu17152546)
Supplement: Supplementary file 1 [file nutrients-17-02546-s001.zip › Supplementary Table 3.pdf]

Supplementary Table S3. Spearman's correlation matrix between lipogenesis-related gene expression in PBMCs from children.

	ATF4	*FTO*	*SREBP1*	*FASN*	*ACACA*
ATF4	1	Rho= 0.494<br>P >0.001	Rho= 0.349<br>P= 0.004	Rho= 0.414<br>P= 0.001	Rho= 0.393<br>P= 0.001
FTO		1	Rho= 0.620<br>P >0.001	Rho= 0.441<br>P >0.001	Rho= 0.545<br>P >0.001
SREBP1			1	Rho= 0.162<br>P= 0.198	Rho= 0.566<br>P >0.001
FASN				1	Rho= 0.132<br>P= 0.291
ACACA					1

SREBP1: Sterol Regulatory Element-Binding Protein 1; *FASN*: Fatty Acid Synthase; *ACACA*: Acetyl-CoA Carboxylase Alpha; *FTO*: Fat Mass and Obesity-Associated Gene; *ATF4*: Activating Transcription Factor 4. Rho values correspond to the Spearman correlation coefficients. P values < 0.05 were considered statistically significant.
